# Editorial: Parasites at the one health interface

**DOI:** 10.3389/fvets.2025.1685326

**Published:** 2025-10-27

**Authors:** Mughees Aizaz Alvi, Hong-Bin Yan, Hussam Askar, Sirikachorn Tangkawattana

**Affiliations:** ^1^Department of Clinical Medicine and Surgery, University of Agriculture, Faisalabad, Pakistan; ^2^State Key Laboratory for Animal Disease Control and Prevention, College of Veterinary Medicine, Lanzhou University, Gansu Province Research Center for Basic Disciplines of Pathogen Biology, Key Laboratory of Veterinary Parasitology of Gansu Province, Key Laboratory of Veterinary Etiological Biology and Key Laboratory of Ruminant Disease Prevention and Control (West), Ministry of Agricultural and Rural Affairs, National Para-reference Laboratory for Animal Echinococcosis, Lanzhou Veterinary Research Institute, Chinese Academy of Agricultural Sciences, Lanzhou, China; ^3^Faculty of Science, Al-Azhar University, Assiut, Egypt; ^4^State Key Laboratory for Animal Disease Control and Prevention, Lanzhou Veterinary Research Institute, Chinese Academy of Agricultural Sciences, Lanzhou, China; ^5^Faculty of Veterinary Medicine, Khon Kaen University, Khon Kaen, Thailand

**Keywords:** One Health, zoonotic parasites, parasitic diseases, surveillance and control, neglected tropical diseases

Relationships among human health, animal health, environmental health, both interrelationships in general and the connection specifically between human and animals, have become particularly important given the emergence of new infectious diseases, changing environmental conditions, and escalating animal-human interactions. Parasitic diseases are examples of the multidimensional nature of the issues encountered on this interface, which often occurs because of complicated ecological, socioeconomic and behavioral factors. The aspects of parasitic and parasitic-vector borne diseases as threats to global health, as well at the interface of One Health are presented with a rich set of contributions varying from field based, surveillance based and specific parasites.

## Framing the One Health agenda through parasite research

In essence, the One Health framework fosters the use of transdisciplinary approaches in order to learn and reduce the threat of zoonoses. The existence of parasites is featured as a direct and indirect disease mediator, and they have been affected by ecological fluctuations, global business, human utilization of the land, wildlife encounters, and climate change. The articles that are a part of this Research Topic highlight the role of parasites as important ecosystem health sentinels and as under-recognized risks to human and animal health.

Echinococcosis is an exemplary reflection of such nuance, through infections of *Echinococcus multilocularis* (a zoonotic highly pathogenic cestode in humans). Japanese investigators offer two articles that examine this parasite in two different perspectives. Kida et al. report a unique example of *E. multilocularis* infection in domestic dog with evidence of gastrointestinal manifestations in Hokkaido, highlighting its hazard and the need to monitor this parasite in companion animal veterinary practices. Simultaneously, Fukui et al. evaluate the ecologic drivers, including vegetation and proximity to urban centers, affecting the density of fox feces (through which the prevalence of parasites can be inferred) in endemic regions thereby connecting wildlife ecology to the monitoring of population health.

Likewise, zoonotic scabies has re-emerged. According to Christiana et al., there is an alarming incident of *Sarcoptes scabiei* infection passed from dromedary camels to humans is posing a risk to the occupational health of pastoral societies and highlights the need to focus on awareness of diagnostics. Such examples indicate the changing boundaries of zoonotic parasitism and the need to have early warning systems that have to be integrated within both the veterinary and human health care systems.

## Expanding the host spectrum: wildlife and domestic interfaces

Parasitological surveillance of the wildlife species is often neglected, yet the species are important in a disease ecology context. Liu, Li, et al. document the existence of *Pentatrichomonas hominis* in the Tibetan antelope- a species that is not only ecologically sensitive but also one that is indicative of the high-altitude ecosystem. This is indicative of the possibility of translocation, adaptation of parasites in other species living on distant habitats enhanced by the overlap of livestock and wildlife and the resultant climatic changes. On a similar note, Gao et al. cite a huge variation in *Enterocytozoon bieneusi* genotypes in wild rodents in three Chinese provinces, establishing the role of reservoir species in maintaining and transmitting parasites in the wild.

There are also other zoonotic nematodes in the form of the zoonotic filarial nematode genera, *Brugia* which crosses surprising boundaries. Infection with *Brugia* sp. has been identified in a captive lion, which is an extension of the known host ranges and pose a risk to zoo personnel and those who handle wild animals (Junsiri et al.). Likewise, Liu, Zhang, et al. revealed the presence of *Giardia intestinalis* in commercial farm raised fur animals in the north of China, including mink, foxes, and raccoon dogs, which indicates that industrialized animal culture forms a facilitator of protozoan spread amongst the animal units. These case studies indicate the immediacy of embeddedness of wildlife, exotic and farm-raised animals disease and health status in the expanded One Health approaches.

## Human-associated parasitism: neglect, burden, and risk factors

Marginalized populations are frequently overrepresented in parasitic diseases. Getie et al. examined the burden of intestinal parasitic infection in food handlers in Gondar City, Ethiopia, and it was found that the outcomes demonstrated high incidence rates and the cause was due to poor hygiene habits and low education levels. In their study, the researchers emphasize the necessity of specific public health education and the regular screening of the employees within the food industry, particularly in low-resource countries. Parallel to these findings, we have Thailand where Maneepairoj et al. addressed helminth infection around forest-proximal waste stations, and examined rodents and murine-related parasites. They determined that ecological degradation, substandard sanitation, and close proximity to humans are common factors which creates conditions promoting cross-species parasites and increase the risk of parasite exposure to humans.

Ullah et al. investigated the prevalence of the *Hyalomma* tick species on livestock in Pakistan. The investigators found the presence of Rickettsiales DNA, pointing to a dual parasitic-vector status, illuminating the possible underestimation of threats to livestock productivity and even human health in arid regions.

## Environmental spillover and water-linked transmission

Water is one of the major channels in the spread of parasitic infections, particularly in pastoral and peri-urban areas. Rafiq et al. investigated *Cryptosporidium* prevalence of goats and local water, linking the infectivity of the pathogen to pollute the environment and sustain infection rates. Their work contributes to the need to introduce combined livestock-water monitoring and protozoa diagnosis enhancement in order to reduce the contamination of the environment.

Investigation of novel treatments for cryptosporidiosis is another important area. Gattan et al. describe their investigation of the anti-cryptosporidial effect of eugenol, a natural compound, through both initial *in vitro* and subsequent *in vivo* evaluations. The positive results support the utility of plant-based and inexpensive therapeutic solutions, particularly, amid antimicrobial resistance growth and the lack of effective treatments of cryptosporidiosis in general.

## Novel therapeutics and predictive computational approaches

Translational dimensions of parasite research can be well illustrated in contributions that discuss the field of drug research, and disease modulation. Rahman et al. used computational biology tools to identify effective inhibitors (ZINC67974679, ZINC67982856, and ZINC05668040) against *Rickettsia felis*, a pathogen causative in the flea-borne spotted fever disease. The combined power of their multiple methods (virtual screening, pharmacokinetic modeling, and docking analysis), illustrate the future direction of parasitic disease research through rapid, non-expensive, and precision based methods.

Fazilani et al. discussed the possible use of artesunate in the therapy of *Babesia microti* infection in mice, as the drug demonstrates strong antiprotozoal activity. This makes it possible to repurpose drugs on neglected parasitic diseases where there are few investments in the pharmaceutical industry.

Within these series of One Health and zoonotic parasites papers, a study evaluating cross-protection, underscores the importance of parasite immunomodulation, El-kady et al. showed that predisposed *Trichinella spiralis* infection may prevent the development of hepatic fibrosis caused by *Schistosoma mansoni*, indicating that immunomodulatory strategies stimulated by the interaction of two organisms may be a promising solution.

## Microbiota and immunological frontiers

Zeng et al., explored an innovative aspect of gastrointestinal parasites and host microbiome. This study evaluated rats infected with *Anisakis pegreffii* and showed that parasitic infection can bias the host's microbial community. The authors discussed the impact of parasitic infection on disease outcomes and the immune system. The potential therapeutics or diagnostics that may emerge can take advantage of these microbiome-parasite interactions and may be based not on eliminating the parasite, but on altering the host's microbial landscape.

## Moving forward: integration, surveillance, and equity

The various studies and their findings included within this Research Topic demonstrate the diversity of biologic complexity of parasite infections related to One Health. Under the One Health movement, there are several main priorities for the future which include the followings:

1. Fortifying surveillance: surveillance should be enhanced, not only between dogs living in our cities and rodents living in our forests, but also on a species and ecosystem level to identify any emerging threats in a timely manner.
2. Cross-sectoral collaboration: parasitology research need to utilize the molecular biology, computational science, social sciences and veterinary epidemiology toolboxes to be able to deliver a complete and multidisciplinary solution.
3. Building capacity in low-resource environments: a large proportion of the biggest disease burdens of parasite are in the locations with the poorest diagnostic capacity. Local laboratories and field-based training, as well as community education, are also of utmost importance for resource investment.
4. Drug innovation and repurposing: although natural compounds continue to achieve pharmaceutical success, nature already has given us a successful drug, eugenol, and repurposing it and similar compounds can develop new drug candidates, as seen with artesunate.
5. Climate and ecological awareness: research needs to be adjusted to the changing parasite ranges due to global environmental changes with the concurrent ecological models and the long-term surveillance have to become important components of the needed analysis.

The contributions to this Research Topic enhance our knowledge about the topic of parasitism at the interface between human, animals, and the environment and help to emphasize the relevancy of the parasitic diseases on the world health. To deal with these aspects, one will need innovation, equity, and collaboration which are the features of the One Health philosophy.

